# Multidimensional Mapping Method Using an Arrayed Sensing System for Cross-Reactivity Screening

**DOI:** 10.1371/journal.pone.0116310

**Published:** 2015-03-19

**Authors:** Sheryl E. Chocron, Bryce M. Weisberger, Hadar Ben-Yoav, Thomas E. Winkler, Eunkyoung Kim, Deanna L. Kelly, Gregory F. Payne, Reza Ghodssi

**Affiliations:** 1 MEMS Sensors and Actuators Laboratory (MSAL), University of Maryland, College Park, Maryland, United States of America; 2 Fischell Department of Bioengineering, University of Maryland, College Park, Maryland, United States of America; 3 Department of Electrical and Computer Engineering, Institute for Systems Research, University of Maryland, College Park, Maryland, United States of America; 4 Institute for Bioscience and Biotechnology Research, University of Maryland, College Park, Maryland, United States of America; 5 Maryland Psychiatric Research Center, University of Maryland School of Medicine, Baltimore, Maryland, United States of America; Wake Forest University School of Medicine, UNITED STATES

## Abstract

When measuring chemical information in biological fluids, challenges of cross-reactivity arise, especially in sensing applications where no biological recognition elements exist. An understanding of the cross-reactions involved in these complex matrices is necessary to guide the design of appropriate sensing systems. This work presents a methodology for investigating cross-reactions in complex fluids. First, a systematic screening of matrix components is demonstrated in buffer-based solutions. Second, to account for the effect of the simultaneous presence of these species in complex samples, the responses of buffer-based simulated mixtures of these species were characterized using an arrayed sensing system. We demonstrate that the sensor array, consisting of electrochemical sensors with varying input parameters, generated differential responses that provide synergistic information of sample. By mapping the sensing array response onto multidimensional heat maps, characteristic signatures were compared across sensors in the array and across different matrices. Lastly, the arrayed sensing system was applied to complex biological samples to discern and match characteristic signatures between the simulated mixtures and the complex sample responses. As an example, this methodology was applied to screen interfering species relevant to the application of schizophrenia management. Specifically, blood serum measurement of antipsychotic clozapine and antioxidant species can provide useful information regarding therapeutic efficacy and psychiatric symptoms. This work proposes an investigational tool that can guide multi-analyte sensor design, chemometric modeling and biomarker discovery.

## Introduction

Blood is the conduit for transporting chemicals throughout the body. These chemical species perform diverse functions such as communication and energetics, and their activities are vital to the body’s effort to respond to threats and maintain homeostasis [1,2]. Thus, information of chemical compounds in blood provides a snapshot of an individual’s health status and consequently, blood tests are routinely used to discern diagnosis, facilitate prognosis and assess therapeutic actions [2–5]. Blood tests are typically performed in a central laboratory facility using common bench-top equipment (*i*.*e*., chromatography, mass spectroscopy) [6], although the need for real-time and frequent monitoring has led to the development of various point-of-care (POC) devices [7]. Such portable sensor systems can access chemical information in blood and be employed on-site by health-care professionals (*i*.*e*., at an office or pharmacy) or at home by a patient or care-giver to provide faster test results and therapeutic interventions [8]. Other benefits of POC testing include fewer unnecessary hospital admissions, better optimized drug treatment, and improved quality of life [8,9]. The most successful example of a POC device that accesses chemical information in blood is the glucose sensor, which is routinely used by diabetics to monitor their blood sugar and guide the administration of insulin [9]. Glucose measurement is enabled by a lock-and-key sensor design whereby selective recognition elements (enzymes) recognize and transduce the chemical signal of glucose into an electrical signal [4,5].

While the glucose sensor has transformed the management of diabetes, it may not be the best paradigm for other applications such as neuropsychiatric disorders, where the specific biomarkers for diagnosing or monitoring these diseases are not completely understood [10–13] and are in turn more difficult to diagnose and manage [14–16]. The state of these disorders may not be exemplified by a single marker but rather by integrated body responses [10–13,16–18]. Investigational tools that allow samples to be analyzed for population commonalities would enable better understanding of these disorders [10,11,13,15,16]. The typical lock-and-key sensor approach employing specific biological recognition elements (*i*.*e*., enzymes) may be impeded by the lack thereof, which creates a major challenge of cross-reactivity among molecular species in the complex sample (*i*.*e*., blood, urine, sweat, saliva) [7,19,20]. New sensing methodologies for mental health management that are based on multi-analyte, multi-sensor platforms can bypass the need for selective biological recognition elements by accounting for cross-reactions present in complex biological fluids.

Schizophrenia exemplifies such complex conditions lacking well-understood blood markers [10–12,15,16,21–23]. Nonetheless, emerging evidence implicates oxidative stress to have psychophysiological significance in schizophrenia among other conditions. For instance, measures of antioxidants have been shown to correlate inversely to worsening symptoms of psychosis [24,25]. Thus, small molecule candidate markers may include biological antioxidants (*i*.*e*., ascorbic acid [26], uric acid [27]). Frequent monitoring of oxidative stress along with antipsychotic medication levels will allow physicians to assess the efficacy and toxicity of the treatment and guide drug titration [28]. The antipsychotic clozapine (CLZ) is a fitting example of the need of frequent monitoring in schizophrenia treatment. It is the most effective antipsychotic for treatment-resistant schizophrenia and its narrow therapeutic range suggests continuous measurement of its blood level [28]. These markers are currently measured at centralized facilities using common bench-top equipment (*i*.*e*., chromatography, mass spectroscopy) [29]. Nonetheless, the iterative nature of this practice requires a faster and more convenient monitoring platform that can be employed at the POC to improve treatment outcomes. Incorporating miniaturizable electrochemical sensors in POC devices for schizophrenia treatment management is well-suited to probe the redox activities of blood samples and can extract information of individual CLZ and antioxidant indicators due to their electroactive nature [29–31]. We previously demonstrated the ability to detect CLZ with a POC device by incorporation of a semi-selective a redox cycling film on electrode surfaces to enable oxidative signal amplification [32,33]. However, in measuring serum samples, electroactive components present in the matrix generated overlapping and potentially inter-dependent signals to the CLZ measurement due to selectivity limitations (*i*.*e*., no biological recognition elements).

Rather than relying on a single semi-selective electrode, a sensor array is a more suitable platform for accessing the broader scope of information available in the complex sample [16,18,22]. For instance, the diagnosis or prognosis may be inferred from a particular pattern of various semi-specific markers [18]. Thus, concepts such as the artificial tongue, which have enabled multi-analyte assessment in complex mixtures by coupling multi-sensor array responses with pattern recognition (*i*.*e*., chemometric) models, can be a more appropriate platform [22,34–38]. This method relies on semi-selective sensors that each provide diverse and synergistic information regarding the markers of interest as well as other variable background signals to create fingerprint patterns for the markers of interest. Quantitative or qualitative models can be applied to obtain either presence/absence information or concentration values [34]. Moreover, advanced models such as artificial neural networks (ANN) are capable of being trained to a certain sample matrix, such as the particular baseline signal of an individual’s blood sample, and can account for the sample matrix changes that may differ from person to person [39]. Thus, the diversity of a sensor array provides multidimensional information that can account for variable cross-reactive species in complex samples.

**Fig 1:**
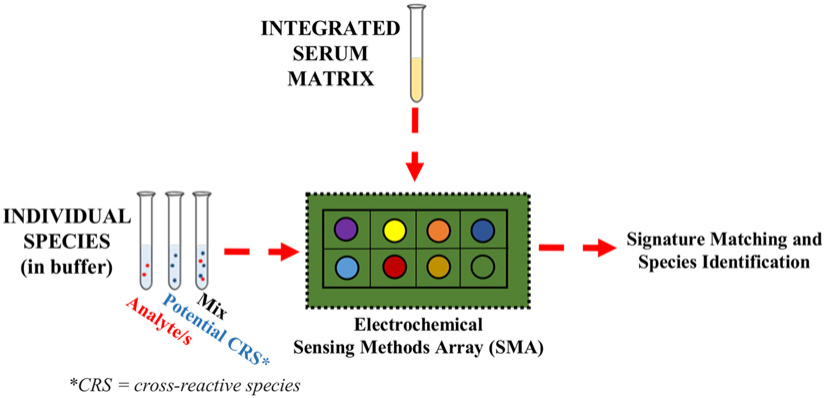
Methodology for Bottom-up and Top-Down Investigation. Schematic representing the systematic methodology for studying the effect of cross-reactive species (CRS) presence on the CLZ measurement by correlating bottom-up and top-down approaches through a sensing methods array (SMA).

We demonstrate an integrated bottom-up and top-down approach that uses an arrayed sensing system to discern and match electrochemical signatures of CLZ and antioxidant species in buffer and serum-based matrices towards the development of a monitoring device for schizophrenia treatment management. In a bottom-up study, a systematic methodology is employed to select, screen and identify electrochemical patterns of endogenous electroactive species in buffer-based solutions. Furthermore, the cross-reactivity of these species with the exogenous CLZ marker is characterized by testing them in simulated mixtures. To capture broader chemical information and enable multi-level comparisons, varying sensing elements are incorporated in an array. The miniaturizable sensing elements consist of differential changes in the input parameters, including changes to the electrical input signal, the sample pH and the sensor material. The sensor array was shown to produce differential responses that provide synergistic information about the chemical species. Lastly, multi-dimensional array responses from bottom-up buffer based studies were compared to a top-down study based on blood serum samples, to discern and match signatures between the two sample matrices, as illustrated in Fig. 1. This methodology provides an understanding of the individual and combined sensor responses of CLZ, uric acid and other components of serum. Broadly, the results outline an investigational tool for identifying signatures of interest (*i*.*e*., cross-reactive species) in any sample, which can be applied to suggest guidelines for targeted sensor design, to chemometric models, or to discover biomarkers.

## Materials and Methods

### Materials

Phosphate buffer saline (PBS, pH 7.4) containing 0.01 M phosphate buffer, 0.0027 M potassium chloride, and 0.137 M sodium chloride was prepared by dissolving concentrated tablets with deionized water. Pooled commercial human serum (from human male AB plasma, originated in the USA, sterile-filtered) were divided into 1 mL aliquots and stored in-20°C conditions. All materials were purchased from Sigma-Aldrich, except urea (Fisher Scientific), L-glutamine (JRH Biosciences), and glycine (Bio-Rad).

### Electrochemical methods

A glassy carbon disk electrode (GCE, 3 mm diameter), a platinum disk electrode (Pt, 2 mm diameter), a platinum wire electrode, and a Ag/AgCl (1 M KCl) electrode were purchased from CH Instruments. Electrochemical tests were performed with a CHI660D potentiostat (CH Instruments) in a three-electrode configuration of either GCE or Pt as the working electrode, platinum wire as the counter electrode and Ag/AgCl as the reference electrode. All potential values presented are written in reference to a Ag/AgCl half-cell potential. The working electrodes were cleaned by successively polishing with 1, 0.3, and 0.05 μm alumina powders and were electrochemically validated before every test. Validation was performed by measuring the ferrocyanide/ferricyanide redox couple using a cyclic voltammetry technique (CV: initial E and final E = 0.19 V, high E = 0.44 V, low E = -0.06 V, positive initial scan polarity, scan rate = 0.2 V/s, 6 sweep segments, sample interval 0.001 V). The redox couple solution consisted of 10 mM PBS, 100 mM sodium chloride, 10 mM ferricyanide and 10 mM ferrocyanide. A differential pulse voltammetry technique (DPV: initial E = 0 V, final E = 0.7 V, increment E = 0.001V, amplitude = 0.05 V, pulse width = 0.2 s, sampling width = 0.0167 s, pulse period = 0.5 s) was employed for elements A–C and F of the sensor array (Table 1). CV (initial E and final E = 0 V, high E = 0.7 V, low E = 0 V, positive initial scan polarity, scan rate = 0.01 V/s, 6 sweep segments, sample interval 0.001 V) was used for elements D–E of the sensor array (Table 1). The pH was changed by adding 1 M HCl or 1 M NaOH under mixing conditions for elements A–C of the sensor array. All buffer-based tests were performed in same-day triplicates and the averages of the signals are shown graphically, such that larger positive responses correlate to higher oxidative current.

**Table 1:** Experimental Conditions for SMA.

Sensing Method	ElectrodeMaterial	ElectrochemicalTechnique	pH
*A*	GCE	DPV, oxidation	7.4
*B*	GCE	DPV, oxidation	**6.5**
*C*	GCE	DPV, oxidation	**8.0**
*D*	GCE	**CV, oxidation, cycle 1**	7.4
*ΔD*	GCE	**CV, oxidation, difference between cycles**	7.4
*E*	GCE	**CV, reduction, cycle 1**	7.4
*ΔE*	GCE	**CV, reduction, difference between cycles**	7.4
*F*	**Pt**	DPV, oxidation	7.4

Experimental conditions for the sensing methods array (SMA), with elements A–F used to generate various sensor responses.

### Serum sample preparation

The frozen serum aliquots were thawed by placing the tube on an ice bed at room temperature. Centrifree Ultrafiltration Devices (# 4104) were purchased from Merck Millipore Ltd and run with 1000 μL serum samples at a centrifugal force of 2000xg for 150 minutes, to remove macromolecules (>30K g/mol) from the sample. A 10 mM CLZ stock solution was spiked into serum prior to measurement to a concentration of 5.6 μM CLZ that accounted for up to 1% of the total sample volume. The pH of serum was found to be about 7.70 and 8.54 before and after molecular weight filtration, respectively. Thus, the pH was adjusted according to Table 1 by adding 1 M HCl for level comparison with the buffer-based results.

### Signal Visualization of Arrayed Responses

MATLAB (R2013b) was used to create heat map representations of the electrochemical responses, using the “imagesc” tool. All signals represented by heat maps were normalized by taking the absolute value and mean-centering, such that larger positive responses correlate to larger oxidative currents for the DPV and the CV’s oxidative sweeps, and larger positive responses in the reductive sweep of the CV correlate to larger reductive currents. Furthermore, the measured electrochemical signal was averaged for 20 mV intervals to assemble the response into 35 groups, and a selected potential range of this smoothed data is shown in the heat maps. The sample array is composed of 8 elements as shown in Table 1: elements A–C represent changes in the sample pH prior to DPV measurement with a GCE working electrode, elements D and E represent the third cycle oxidation and reduction signals from CV measurements with a GCE working electrode, elements ΔD and ΔE represent the difference between the first and third CV cycles of elements D and E with positive values representing an increase of the electrochemical current between cycles, and element F represents the DPV response using a Pt working electrode. Moreover, for the analysis of elements D–F, the electrochemical signal in the presence of the analytes was subtracted by the signal of the buffer solution in the absence of the analytes. Electrochemical peaks on the heat maps are outlined black boxes to highlight the SMA signatures. Simplified heat maps, where only the outlined peaks are shown, are used for overlaying the signatures of several samples such that they can be more conveniently compared. Raw signals not shown here are summarized in the supplemental information.

## Results and Discussion

### Bottom-Up Approach

**Fig 2:**
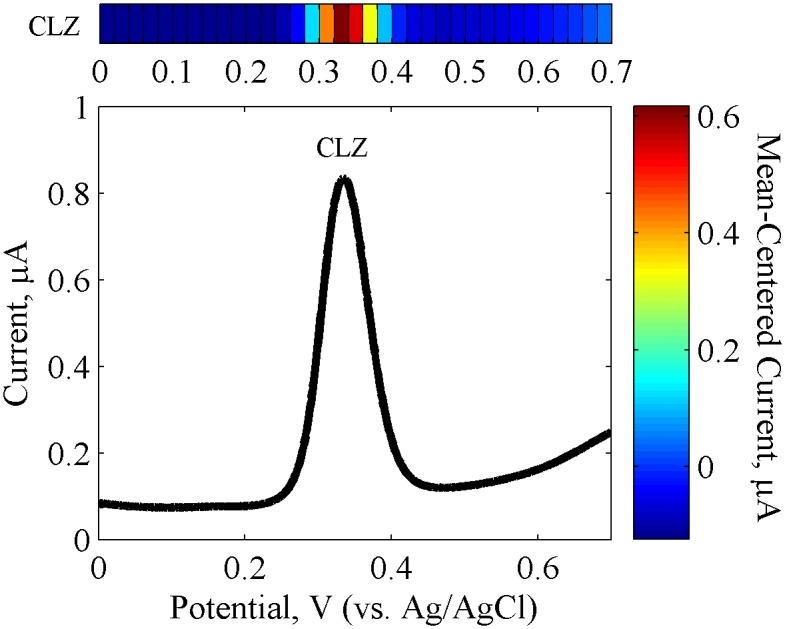
Electrochemical CLZ Response. Differential pulse voltammetry (DPV) and heat map representation of 5.6 μM CLZ in PBS (pH 7.4) using GCE. Signal response represents an average of triplicate measurements.

**Electrochemical Signatures.** DPV scans contain two major components that represent the signatures of an electroactive species, its oxidation potential (*E*_*p*_) whose location varies according to the specific species’ energetics, and its oxidation current (*I*_*p*_) which varies according to the species concentration. For instance, a well-defined CLZ oxidation *I*_*p*_ is seen at an *E*_*p*_ of 0.336 V in buffer (GCE, pH 7.4) as seen in Fig. 2, consistent with the literature [29,40]. This electrochemical response represents one signature of CLZ. And the peak information can be mapped onto a heat map, as shown in Fig. 2, to facilitate comparison across various samples or sensors.

**Screening of Cross-Reactivity with Serum Components.** From all the endogenous (naturally-present) components that make up the complex serum matrix, 17 species were chosen for this study based on the following criteria: (1) common antioxidants [23–26], (2) high abundance [41,42], and (3) known interferences in similar sensors, as shown in Table 2. The latter includes electrochemical sensors for other CLZ sensing schemes as well as other analytes sensed in the measured potential range of 0–0.7 V [4,5,43–49]. Individual species were systematically screened for electroactivity. These criteria can be further individualized for other applications depending on the sensor and analyte of interest. Only four components, aside from CLZ, were shown to be electrochemically active (Table 2): uric acid (UA), L-cysteine (CySH), ascorbate (AA) and oxalic acid (OA) (S1 Fig.). One common theme among the former three species is their antioxidant properties [50–52], with UA accounting for a majority of antioxidant capacity [26], suggesting that antioxidant measurement is amenable to electrochemical sensing assays.

**Table 2:** Cross-Reactivity Screening.

		*Cross-ReactiveSpecies*	*Mixture = Cross Reactive Species + Clozapine*			
	Interfering Species(Upper Concentration, μM)	${\bar{E}}_{p,CS}$ (V)^a^	$\Delta {\bar{I}}_{p,mix}$ (μA)^b^	p-value of $\Delta {\bar{I}}_{p,mix}$ ^c^	$\Delta {\bar{E}}_{p,mix}$ (V)^d^	p-value of $\Delta {\bar{E}}_{p,mix}$ ^c^
*Analyte*	Clozapine (5.6)	0.336	N/A	N/A	N/A	N/A
*High Concentration Antioxidants*	**Uric Acid (410)**	**0.245**	**5.0160**	**9.02x10** ^**–7**^	**-0.0843**	**1.69x10** ^**–6**^
	Ascorbate (40)	< 0.08	-0.0111	0.8571	0.0007	0.8309
	**L-Cysteine (60)**	**0.347**	**-0.3175**	**0.0068**	**-0.0487**	**2.09x10** ^**–5**^
*Most Abundant Organic Metabolites*	D-Glucose (6100)	N/A	-0.07423	0.1858	-0.0037	0.2505
	Urea (9000)	N/A	-0.0699	0.3135	-0.0047	0.0325
	ATP (3000)	N/A	-0.0206	0.7888	-0.0030	0.4557
	DL-Glyceraldehyde (1500)	N/A	-0.0571	0.5287	-0.0003	0.9465
	L-Lactic Acid (2400)	N/A	-0.0832	0.1443	-0.0030	0.4103
	L-Glutamine (670)	N/A	-0.0362	0.4611	-0.0037	0.2913
	L-Alanine (410)	N/A	-0.0574	0.3265	-0.0037	0.0254
	Glycine (282)	N/A	-0.0680	0.2230	-0.0040	0.0705
	L-Lysine (217)	N/A	0.0661	0.1224	-0.0020	0.1447
*Additional Known Interference in Similar Sensors*	L-Valine (276)	N/A	0.0120	0.8406	0.0023	0.2564
	L-Arginine (140)	N/A	-0.1052	0.1357	-0.0053	0.3671
	L-Glutamic Acid (150)	N/A	-0.0139	0.7502	0.0013	0.4918
	L-Methionine (40)	N/A	0.0030	0.9520	0.0013	0.6918
	Oxalic Acid (22)	~ 0.35	-0.0492	0.5661	0.0020	0.4885

Electrochemical oxidation peak values of selected serum species (cross-reactive), and statistical analysis of cross-reactivity when CLZ is tested in mixture with chosen serum species, as a method for screening significant cross-reactions.

^a^ ${\bar{E}}_{p,CRS}$ represents the mean peak current of the cross-reactive species (CRS) alone, if there is observable oxidation within the testing range.

^b^ $\Delta {\bar{I}}_{p,mix=}\left|{\bar{I}}_{p,mix}\right|-\left|{\bar{I}}_{p,CLZ}\right|$, where ${\bar{I}}_{p,i}$ represents the mean peak current of either CLZ or the mixture of CLZ and CRS.

^c^ The p-value is the result of a one-way ANOVA test between CLZ-only and simulated mixture peaks. The corresponding peak in the mixture was determined as the largest detectable peak.

^d^ $\Delta {\bar{E}}_{p,mix=}\left|{\bar{E}}_{p,mix}\right|-\left|{\bar{E}}_{p,CLZ}\right|$, where ${\bar{E}}_{p,i}$ represents the mean peak potential of either CLZ or the mixture of CLZ and CRS.

Additionally, cross-reactivity between the endogenous species and the CLZ exogenous species was assessed in simulated mixtures of the two species at their upper physiological concentrations (Table 2) [41,42], as suggested in the CLSI EP7-A2 guidelines [19]. The electrochemical peaks found in simulated mixtures (individual endogenous species + CLZ) were compared to CLZ-only responses to assess the effect of the simultaneous presence of the two components on the CLZ response. These synthetic mixtures are referred to as simulated because they represent interactions only between two-component mixtures, a step toward identifying species interactions in complex biological fluids. Thus, it does not, for example, consider the simultaneous presence of additional species that may lead to further cross-reactions or effects from sample stability, preparation and storage. The cross-reactivity is represented in terms of the signal difference of the mixture and the CLZ-only solution as well as the corresponding p-values obtained from a one-way ANOVA test between the two sample populations, as seen in Table 2. The cutoff significance level was chosen to be α = 0.1 for the peak current, and α = 0.01 for peak potential since the latter has intrinsically less variation. UA and CySH were found to have significant cross-reactivity with CLZ with respect to both peak current and peak potential, as shown by the p-values in Table 2. Thus, their interaction with CLZ was further studied in the following section.

**Multi-Modal Sensing Array.** Creating a sensing methods array (SMA) enables multi-dimensional signal mapping and facilitates the determination and comparison of characteristic signatures across different conditions. The 8 various sensing elements composing the SMA are shown in Table 1 and are further described below. First, the SMA was validated using a CLZ sample and then it was applied to UA and CySH species individually and in simulated mixtures with CLZ in order to characterize their cross-reactivity.

The design of the sensing methods was chosen to yield differential responses while minimizing complexity. As shown in Table 1, they comprise of only one variable change compared to a control (sensing element A), and include changes of the input potentiostat settings, the pH, and the electrode materials. These design choices are further explained below, and produce diverse signatures as shown by its application to clozapine measurement.

**Electrode Material.** The properties of the electrode material can affect molecular interactions between the electrode and species being measured, and in turn can affect the sensor semi-selectivity across different species [53]. For instance, attraction/repulsion forces between the surface structures of the electrode and the species in solution can affect reaction kinetics [54,55]. In regards to the GCE, studies have shown that species containing amine groups can form a carbon-nitrogen linkage with GCE after oxidation of the amine group to the corresponding cation radical [56,57]. The level of substitution of the amine plays a major role in the electrochemical oxidation kinetics as well as in the GCE linkage. For instance, tertiary amines have been found to have most facile oxidation but an undetectable degree of binding to the GCE, likely because of steric hindrance. Furthermore, secondary and primary amines have the least facile oxidation but are seen to form carbon-nitrogen linkages with GCE [57]. CLZ oxidation on GCE is shown in Fig. 2 with a clean background signal.

**Fig 3:**
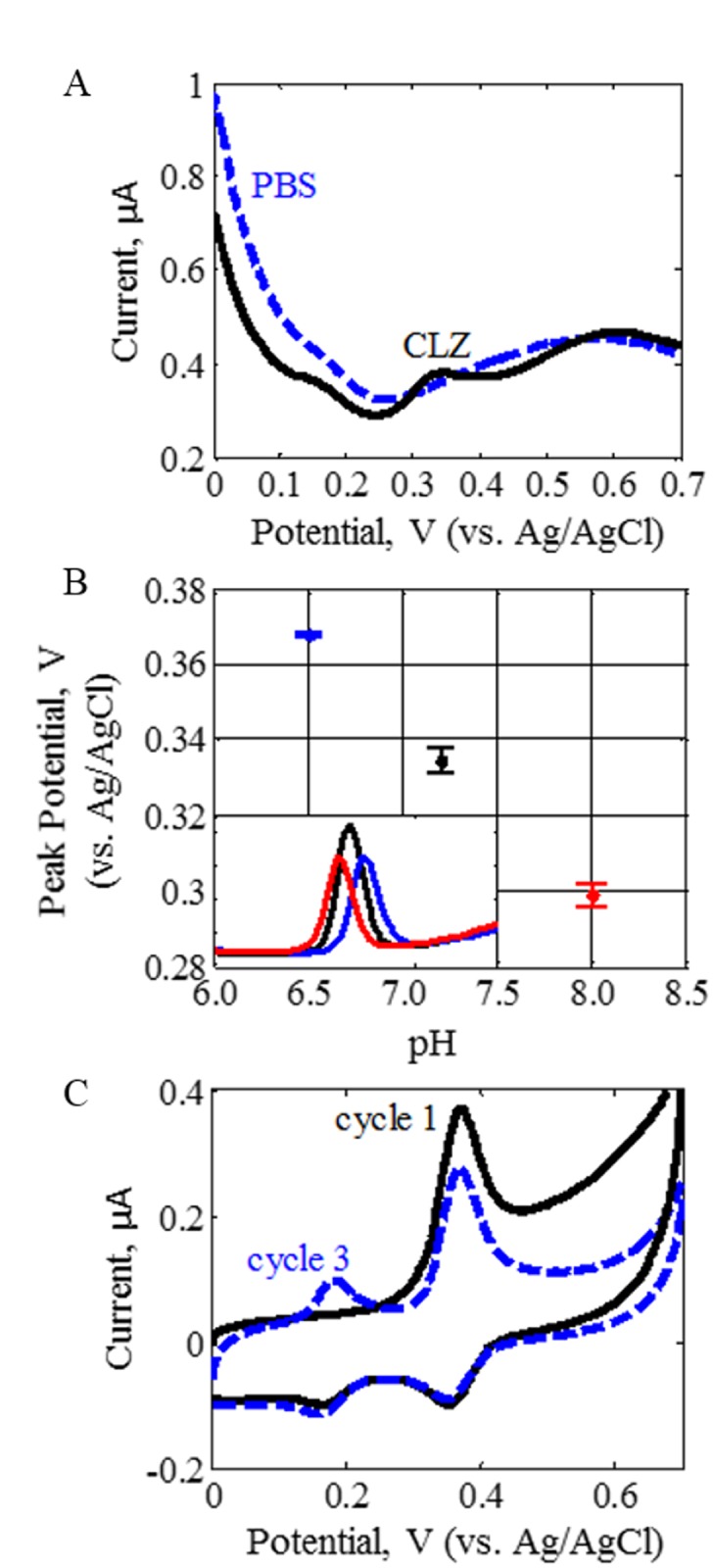
Electrochemical Response of the SMA Components. The SMA responses of 5.6 μM CLZ in PBS are shown across the various sensing element. (A) CLZ DPV signal with the Pt electrode (element F) is compared to background reactions of PBS at pH 7.4, (B) CLZ DPV signals with shifting peak potential for pH 6.5, 7.4 and 8.0 (elements A–C) using a GCE, and (C) CLZ CV signal at pH 7.4 using GCE for cycles 1–3 (elements D–E). The A–F annotations refer to the various elements in the SMA (Table 1). The insert shows the linear trendline of peak potential dependent on pH. Each curve represents the average of triplicate measurements.

A metallic electrode material, Pt, undergoes different surface reaction processes (adsorption, kinetics, surface functionalization) compared to the carbon-based GCE [58,59]. As shown in Fig. 3a, the Pt electrode has background DPV peaks near 0 V and 0.57 V in the PBS solution. In the presence of CLZ, an additional DPV peak was seen at 0.33 V, which does not overlap with the background signals (Fig. 3a). Due to the presence of background reactions, we suspect that changes in the sample matrix may not only affect the CLZ peak but also the background oxidation processes. Thus, the background signal is advantageous in this type of sensor array because it can account for environmental changes and matrix effects that may not be detectable with a GCE. Additionally, combining Pt and carbon electrodes has previously been shown to add orthogonal information in an array of several electrode materials [60].

**pH Changes.** Changes to the solution pH have the ability to affect the oxidation peak potential, E_p_ (V), which depends on the pH and the specific oxidation properties of the species according to the Nernst equation:

$$E_{p}=E^{o}-\frac{2.3RTm}{nF}pH$$ (1)

with the potential at standard conditions *E* ^*o*^ (V), gas constant *R* (J K^-1^ mol^-1^), temperature *T* (K), the ratio of protons *m* to electrons *n* transferred during the redox reaction, and Faraday constant *F* (C mol^-1^). The Nernst equation assumes a reversible redox reaction, fast kinetics, and the concentrations at the electrode surface to be in equilibrium with the potential. These assumptions are not completely met for the semi-reversible, slow reaction of CLZ but serves as a general trend. Fig. 3b shows the linear correlation of CLZ oxidation *E*_*p*_ at varying pH, with a slope of 0.048 V/pH and intercept value of 0.682 V as outlined in Table 3. The behavior of the *I*_*p*_ (Fig. 3b) is seen to increase between the pH 6.5 and 7.4 interval and decrease between pH 7.4 and 8.0, a trend similar to that seen by Huang et al [61]. This could be attributed to several factors. One possibility is changes to pH-dependent functional groups within the GCE surface that can lead to differences in attraction/repulsion forces, reaction kinetics or reaction mechanisms. An additional factor is the effect of pH on the solubility of CLZ, which is least soluble in basic pH.

**Table 3:** pH Dependence According to Nernst Equation.

	Species	Peak Potential(at pH 7.4), V	Slope, V/pH	Intercept, V	R^2^
*Individual Species in PBS*	Clozapine	0.336	0.048	0.682	0.987
	Uric Acid	0.245	0.046	0.579	0.984
	L-Cysteine	0.347	0.141	1.400	0.987
*Mixtures in PBS*	Uric Acid+Clozapine	0.245	0.047	0.592	0.997
	L-Cysteine+Clozapine	0.284	0.059	0.723	0.982
*Un-Spiked Deproteinized Serum*	(suspected) Uric Acid	0.248	0.051	0.610	0.894
*CLZ-Spiked Deproteinized Serum*	Clozapine	0.312	0.0349	0.569	0.997
	(suspected) Uric Acid	0.248	0.0367	0.5063	0.860

Dependence of peak potential on pH based on the Nernst equation ($E_{p}=E^{o}-\frac{2.3RTm}{nF}pH$), with the peak potential *E*_*p*_ (V), potential at standard conditions *E°* (V), gas constant *R* (J K^-1^ mol^-1^), temperature *T* (K), the ratio of protons *m* to electrons *n* transferred during the redox reaction, and Faraday constant *F* (C mol^-1^). The slope values correspond to the ratio of protons to electrons involved in oxidation reactions and the intercept is an estimate of the standard oxidation potential *E°*. Triplicate measurements were carried out for PBS-based solutions and duplicate measurements for serum-based solutions.

**Electrochemical Technique.** In addition to DPV, a different electrochemical method with the potential to incorporate additional useful information was chosen. While DPV is a pulsing technique that is swept across potentials in one direction, cyclic voltammetry (CV) is a (non-pulsing) linear sweep performed in cycles spanning the anodic and cathodic potential directions. Applying CV can lead to oxidation as well as reduction peak responses, and multiple CV cycles allow dynamic visualization of byproducts formed over time. Van Leeuwen et al. showed the following scheme (equation 2), where CLZ can be reversibly oxidized and reduced. Due to the instability of reaction products, byproducts are formed, some of which possess redox behavior. The electroactive byproduct is thought to correlate to hydroxylated CLZ derivatives [40]. Fig. 3c shows the oxidation and reduction peaks of the CLZ cyclic voltammogram near 0.34 V as well as the appearance of oxidation and reduction of the CLZ byproduct near 0.17 V. Furthermore, the peak changes across consecutive CV cycles demonstrates a decrease of the primary CLZ oxidation peak and an increase of the byproduct oxidation peak with cycles (time).

$$CLZ\overset{}{⇌}CLZ^{+1}+2H^{+1}+2e^{-1}\overset{}{\to }Byproduct\overset{}{⇌}Byproduct’$$ (2)

**SMA Signatures of Individual Endogenous Species.** The signatures of UA and CySH are similarly characterized using the SMA to determine their trends across the array for comparison with CLZ signatures and later, for comparison with serum-based responses.

**Fig 4:**
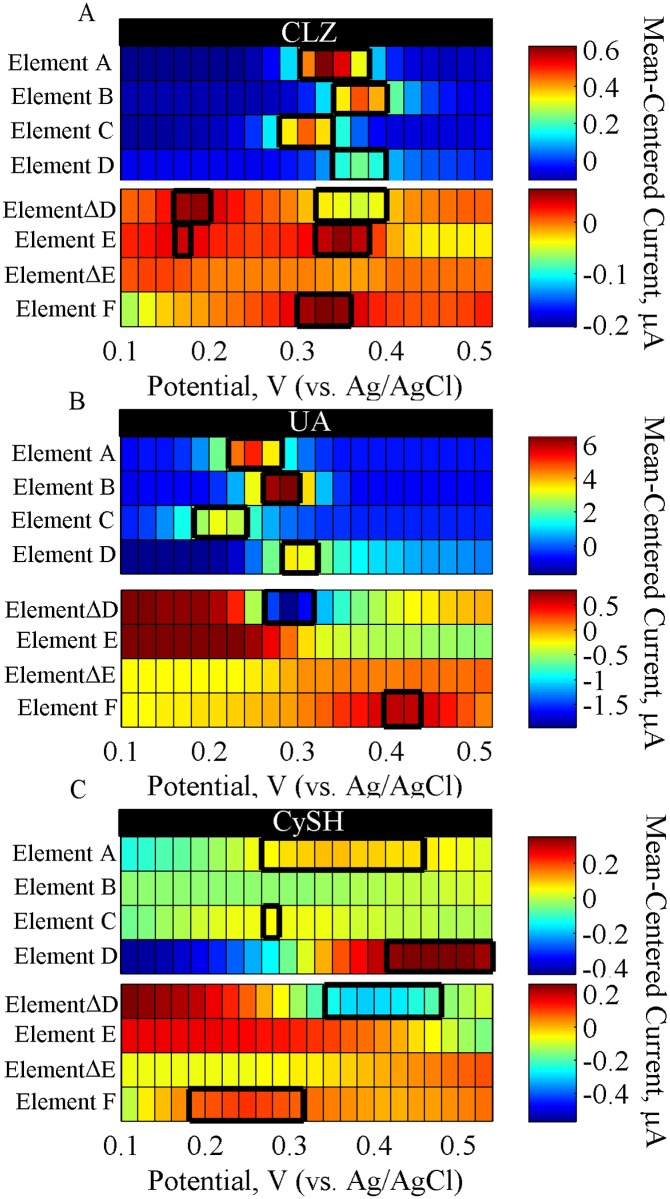
SMA Response of Individual Species in PBS. Heat map representation of electrochemical responses of the sensing methods array for (A) 5.6 μM CLZ, (B) 410 μM UA, and (C) 60 μM CySH tested individually in PBS buffer. The A–F annotations refer to the various elements in the SMA (Table 1), and the peak signatures are outlined in black rectangles. Each response represents the average of triplicate measurements. Note that two scales are used in each heat map to enhance visualization.

UA has a characteristic oxidation peak at 0.245 V in element A as shown in Fig. 4b. Elements A–C illustrate how the peak potential shifts with varying pH between 6.5 and 8.0 according to equation 1, with a trend shown in Table 3. The fitted slope value of 0.046 V/pH is similar to that of CLZ (0.048 V/pH), which suggests a similar ratio of proton to electron produced during the oxidation reactions of these species (equation 1). Nonetheless, the intercept value, representative of the *E* ^*o*^, provides a distinguishable parameter between the two species as shown in Table 3. Notably, the UA produces the highest response at pH 6.5 (element B) and lowest at pH 8.0 (element C). Elements D–E (Fig. 4b) represent the CV signatures of UA. Element D shows the oxidation peak of UA positioned near 0.30 V, and element ΔD demonstrates a decrease in the UA peak over time, suggesting a large consumption of UA throughout cycling. This behavior likely arises due to the electrochemically irreversible behavior of UA, seen by the lack of reduction peaks (element E). Lastly, the UA response at a Pt electrode (element F) shows a drastic shift of 0.18 V in its *E*_*p*_ position between the GCE and Pt electrode materials, demonstrating that different electrode processes can be achieved by varying the electrode material. This trend is a useful signature because it will enable verification of suspected UA peaks in serum by verifying the trend of relative *E*_*p*_ positions between GCE and Pt.

Fig. 4c shows the characteristic signatures of CySH across the SMA. The response at element A shows a wide oxidation peak of CySH at 0.347 V. This peak is suggested to correspond to a 2-step reaction of CySH’s thiol group, which yields CyS^-^ and subsequently disulfide CyS-SCy [62,63]. Thus, an important physiological role of CySH is the formation of disulfide bonds between polypeptide chains, contributing to protein folding [62], which are formed via an oxidation reaction that yields CySH intermolecular links [64].

Elements A–C illustrate large differences in oxidation *E*_*p*_ and *I*_*p*_ across the pH range (Fig. 4c), with a distinguishable wide peak observed at neutral pH. At higher pH (8.0), this peak shifts 0.08 V to lower potentials, and at lower pH (6.5) it becomes ill-defined. Thus, CySH oxidation depends on pH as outlined in Table 3, with a slope of 0.141 V/pH and intercept value of 1.400 V, which are much higher than those measured for CLZ or UA. This suggests characteristic differences in electrochemical processes among the species. Elements D–E show the CV signatures of CySH. A CySH oxidation is seen in the CV as a large peak near 0.48 V (element D), with a magnitude that decreases over time as seen by element ΔD. Similar to UA, this behavior suggests consumption of CySH at the electrode throughout cycling. Moreover, no reduction reactions are detected in the CV, as seen by the lack of peaks in elements E—ΔE (Fig. 4c). Lastly, a DPV response using the Pt electrode (element F) demonstrated a 0.07 V shift of CySH oxidation to higher E_p_ compared to the GCE control. Moreover, an additional peak appears near 0.15 V, but no conclusions can be drawn about this peak since the response between 0 and 0.2 V also correspond to background oxidation processes of the background PBS response, as seen in Fig. 3a.

**SMA Signatures of Simulated Mixtures.** Simulated mixtures of CLZ in the presence of UA or CySH were characterized with the SMA in order to elucidate the effect of their simultaneous presence a further mimic their combined behavior in complex serum solutions.

**Fig 5:**
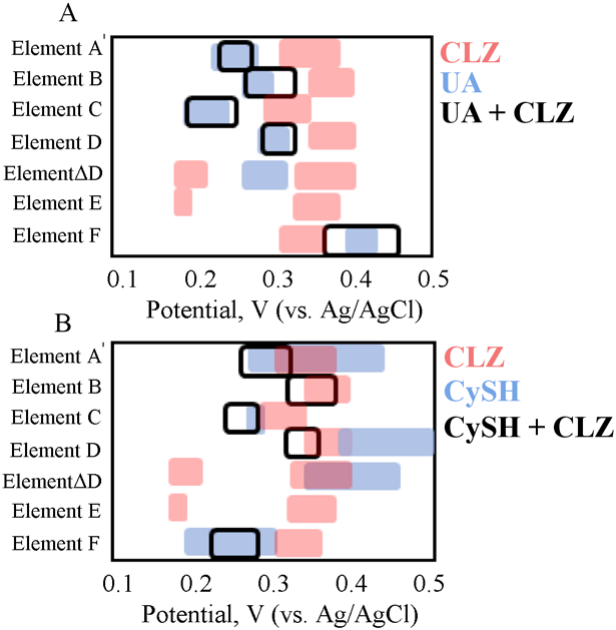
SMA Response of Simulated Mixtures in PBS. Simplified heat map representation of electrochemical responses of the sensing methods array for 5.6 μM CLZ in a mixture with (A) 410 μM UA, and (B) 60 μM CySH in PBS buffer (black outline), compared to their individual counterparts (shaded). The rectangular shapes represent the signatures derived from the heat maps of each of the species such that they can be overlaid. The A-F annotations refer to the various elements in the SMA (Table 1).

Fig. 5a illustrates the signatures of simulated UA/CLZ mixtures as a simplified heat map. The response of element A has a single DPV oxidation peak positioned at the characteristic UA oxidation location (as seen by the blue-shaded outline). This peak has a near 5-fold higher amplitude compared to the CLZ peak *I*_*p*_, and the CLZ signature is masked in the mixture as seen by the lack of mixture signals near the red-shaded outline of the CLZ peak. Similar behavior is seen in the CV oxidation response (element D). Additionally, at varying pH, the linear trend closely follow the parameters seen for UA (Table 3). Even at pH 8.0 (element C), where UA exhibits the lowest signal amplitude, the CLZ peak remains masked. The CV reduction peaks of CLZ in element E were also masked in the presence of UA. This finding corresponds with previous studies, where the interference from high concentrations of UA for electrochemical CLZ measurements was reported [61]. This mixture response suggests an interaction where the UA may be preventing or masking CLZ’s signature oxidation peak, and the behavior may be related to UA’s potent antioxidant activity. Another suggestion may be the formation of linkages between oxidized UA and the GCE surface due to the presence of high concentration of UA’s secondary amines, which can foul the electrode surface (as described for GCE previously) and thus decrease the current response of subsequent electrode reactions. The response of the Pt electrode in element F shows a change in the width of the UA signal in the presence of CLZ, but again the UA signal dominates over that of CLZ. Nonetheless, even when the CLZ is shown to be masked by the upper UA concentration in the simulated mixtures, complex solutions may have different integrated responses in the simultaneous presence of other species. This is further studied in the Top-Down section below.

Fig. 5b shows the signatures of simulated CySH/CLZ mixtures. While the individual peaks of CySH and CLZ have similar *E*_*p*_ values around 0.35 V (for element A) as seen by the overlapping shaded signals in Fig. 5b, the mixture DPV response contains only one major peak located at 0.284 V, with a 0.62-fold change in *I*_*p*_ compared to CLZ individually. In parallel, a small additional peak near 0.472 V (at pH 7.4) appeared in the mixture (S4 Fig.). Because this peak was not observed in either CLZ or CySH individual signals, a cross-reaction between these two components is likely, and can also explain the decreased *E*_*p*_ and *I*_*p*_. Van Leeuwen et al. showed that the CySH-containing glutathione species can react with activated CLZ to form CyS-CLZ adducts, which are electroactive at higher potentials [40]. Thus, the latter peak shown at 0.472 V may be the electroactive product of the Cys-CLZ cross-reaction. Moreover, the pH dependency of the major peak of the mixture follows a trend shown in Table 3, with a slope of 0.059 V/pH that is closer to that of CLZ (0.048 V/pH) than to that of CySH (0.141 V/pH). Thus, this peak is likely governed by the CLZ rather CySH reaction kinetics. And the additional peak near 0.472 V also has a pH-dependent slope of 0.0432 V/pH closely resembling that of CLZ. The CV reduction response in element E shows that the CLZ reduction peak missing. This behavior along with the decreased peak current may be explained by the CyS-CLZ production, which would increase the rate of CLZ consumption in a reaction with CySH and reduce CLZ’s reduction reaction. Lastly, the response of the Pt electrode in element F illustrates signatures that resemble the CySH trend and mask the CLZ peak. This sensing element is seemingly more selective to CySH than to CLZ.

### Top-Down Approach

Serum complexity makes comparison of buffer and serum based signal responses challenging due to matrix effects. The matrix effect refers to the differences in chemical (simultaneous presence of a combination of interfering species, pH) and physical (viscosity, electrostatic forces, temperature history) properties of the matrix compared to the calibration solution (PBS), that may lead to alterations of the signal [19,65]. Additionally, changes in matrix composition as well as interactions between matrix components and the analyte, need to be considered. One can account for some of the matrix property differences (*i*.*e*., adjusting serum pH, choosing a buffer with similar ionic properties, and predicting the effect of preparation), but unaccounted matrix effects are likely to affect the response where highly complex cross-reactive behavior is expected. Thus, the top-down approach is the direct measurement of complex solutions such as blood serum, and accounts for the combined behavior of the analytes in the presence of other matrix components and conditions. Previously measured simulated mixtures (of CLZ + individual cross-reactive component) resemble serum-based solutions more closely than individual species because their response accounts for some combined cross-reactive behavior. Nonetheless, it only accounts for the cross-reactions of two species at a time. Thus, comparison between the signatures seen in bottom-up simulated mixture studies with top-down serum-based studies is important to account for matrix effects.

**Fig 6:**
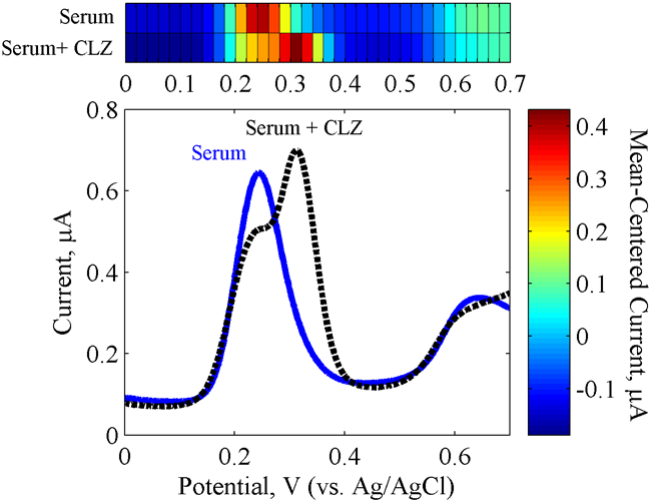
Electrochemical Serum Response. Differential pulse voltammetry (DPV) of serum with and without 5.6 μM clozapine using sensing element A, and heat map signature representation. All solutions were tested using GCE, and represent an average of duplicate measurements.

**Single-Sensor Analysis.** The electrochemical activity of un-spiked and CLZ-spiked serum was tested first with a single GCE sensor (element A) with the DPV technique at pH 7.4. Fig. 6 shows the measured voltammograms and corresponding heat map representing the response of serum with and without CLZ. A predominant peak present in the DPV response of the serum background was observed at 0.248 V and another peak at 0.647 V. Comparing these signatures with the corresponding buffer-based signatures, the first peak can be matched to the characteristic oxidation potential of UA (0.245 V). While about 6–8 times higher *I*_*p*_ magnitudes were expected for the UA signal due to its high physiological concentrations, species degradation may have occurred during sample preparation, transportation and preservation [66,67]. Moreover, the second peak at 0.647 V did not match to any of the interfering species studied in previous sections. The latter may be a result of signal changes in the simultaneous presence of several interfering species or a result of endogenous species not assessed in this study. Lastly, current peaks that can be matched to CySH peak signatures (0.347 V) were not observed in the serum response. This species may not be detectable in serum due to its low concentrations, overlap with the larger UA peak, or potential cross-reactions with other endogenous components during storage and handling. In the presence of CLZ, a third peak at 0.312 V appears. This peak is near the location of the CLZ peak measured in PBS and has about 0.78-fold decreased *I*_*p*_ compared to the buffer-based response. Furthermore, the same peaks found in the un-spiked serum response remained present in the spiked serum response, with a slight decrease of the suspected UA peak. This decrease may be due to a cross-reactive dependencies between CLZ and UA.

**Fig 7:**
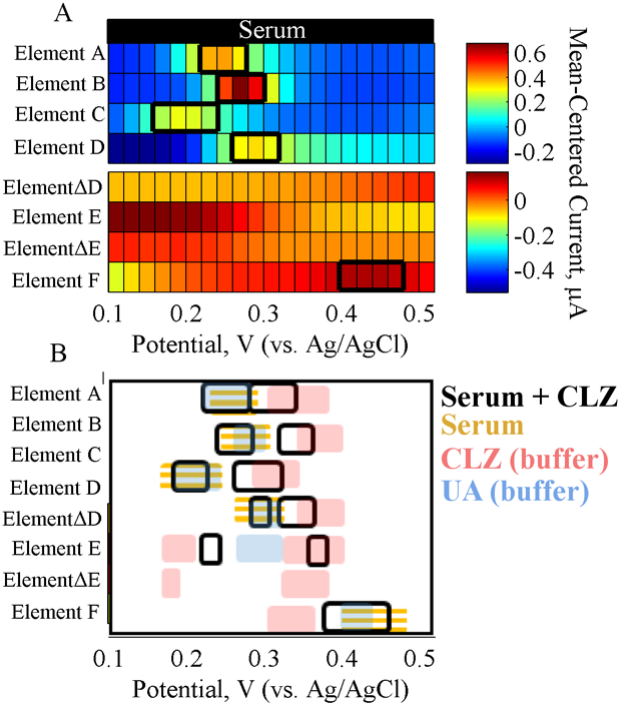
SMA Response of Serum. (A) Heat map representation of electrochemical responses of the SMA for serum, with signatures highlighted in black outlines. (B) Simplified heat map of the electrochemical responses of the SMA showing only the outlined signatures of serum spiked with 5.6 μM CLZ, overlaid with outlined signatures of un-spiked serum (orange lines) and well as CLZ and UA in buffer (shaded). The simplified heat maps illustrate the overlapping signatures such that they can be matched across the samples. The A-F annotations refer to the various elements in the SMA (Table 1).

**SMA Analysis.** The SMA was applied to serum-based tests to demonstrate the advantages of applying a multi-dimensional sensor array to characterize un-spiked and CLZ-spiked serum responses, and match their responses to the bottom-up studies of buffer-based samples. Fig. 7 shows the heat maps representing the integrated SMA response of the serum samples in order to facilitate comparison with buffer-based signatures.

The responses of elements A–C illustrate the change in serum electrochemistry with varying pH at the GCE in Fig. 7. In the un-spiked serum, the suspected UA peak (at 0.248V, element A) follows a pH response trend matching that of the individual UA response, as seen in Table 3. Thus, this results further points to the likelihood of this peak representing UA oxidation since the fitted values corresponding to the proton to electron production ratio and the standard potential was similar to that of UA oxidation. Interestingly, the pH trends for the two overlapping peaks seen in the CLZ-spiked serum demonstrate a consistent decrease in slope and intercept values of the two peaks compared to the buffer-based trends, shown in Table 3. This discrepancy in the pH trends may be caused by cross-reactions between UA and CLZ in the presence of other interfering species in this complex mixture, and further illustrates the dependence of analyte measurement on other components of the sample. Notably, the highest resolution and differentiation of the two major peaks was seen at pH 6.5 (element B), as seen in Fig. 7b.

The response of elements D–E illustrate the CV responses and include Elements ΔD—ΔE which represent the difference in current over a period of three CV cycles (or time). As seen in Fig. 7, the CV oxidation response of serum in element D is similar to the DPV response in element A. However, the changes in the CV oxidation response over time (element ΔD) further points to a larger decrease of the suspected UA peak compared to a smaller decrease seen for the CLZ peak, as shown in S7 Fig. Thus, this difference provides an additional signature for distinguishing the two species. Elements E and ΔE provide information about reduction reactions, which was previously shown to be another differentiating factor between UA and CLZ because CLZ has a reduction peak whereas UA does not. As can be seen in the response of un-spiked serum (Fig. 7a, element E), no reduction peaks can be distinguished. Even when CLZ was spiked into serum, no reduction peaks were detectable (Fig. 7b, element E), which matches the behavior seen in the simulated mixtures of CLZ with UA (Fig. 5a).

The response of element F corresponds to the Pt electrode response. As seen in Fig. 7a (element F), a major peak near 0.430 V was observed for the un-spiked serum sample. The location is drastically different compared to the background serum peak in element A at the GCE. This peak shift across the two electrodes matched the behavior seen for the individual UA buffer-based response, further corroborating this peak likely corresponds to UA. When CLZ was spiked into serum, only one peak remained in the response near the peak seen for un-spiked serum, although it was shifted toward lower potentials. Similar behavior was seen in the buffer-based UA/CLZ simulated mixture response, which was hypothesized to belong to an integrated UA/CLZ response. This sensing element suffers from reduced resolution compared to the GCE, however, it shows a characteristic signature of UA that changes in the presence of CLZ. Thus, this response can provide an integrated measure of multiple species.

## Conclusions

A novel integrated bottom-up and top-down approach was employed using an arrayed sensing system to discern and match signatures in buffer and complex solutions. Multidimensional signatures of individual species and their simulated mixtures were collected using a sensing methods array (SMA) to elucidate inter-dependencies. By applying this investigational tool to the model application of serum-based measurement of CLZ and antioxidant analysis, the bottom-up study identified UA and CySH as being electroactive and cross-reactive with the antipsychotic CLZ during measurement. Using the SMA for top-down studies of the complex serum matrix, UA and CLZ signature trends were matched to bottom-up results across the elements of the array. Some differences in the signatures of these species in buffer and complex solutions were observed and attributed mainly to additional molecular cross-reactivity and integrated matrix effects. These results further highlight the advantages of using an arrayed sensing system for mapping complex solutions as well as the challenges of inter-dependence between the analyte and other matrix components. We show that collecting broader information enabled discerning of cross-reactions in simulated and complex serum matrix. The ultimate sensor design is envisioned as an integrated SMA platform incorporated with savvy pattern recognition (*i*.*e*., chemometric) data processing that takes to account the inter-dependence of cross-reactive species for measurement in complex samples. Furthermore, this methodology can be applied for the investigation and simultaneous measurement of various disease-related markers, for the assessment of various sample conditions or treatments, or for sensor characterization and optimization.

## Supporting Information

S1 Fig Response of Some Electroactive Serum Components. Differential pulse voltammetry (DPV) of 60 μM L-cysteine, 410 μM uric acid, 22 μM oxalic acid, 40 μM ascorbate, and 5.6 μM CLZ (pH 7.4) in PBS at the GCE. Signal response represents an average of triplicate measurements.
(TIF)

S2 Fig Electrochemical Response of UA with the SMA Elements. The SMA responses of 410 μM UA in PBS are shown across the various sensing element. (A) UA DPV signals at pH 6.5, 7.4 and 8.0 (elements A–C) using a GCE, (B) UA CV signal at pH 7.4 using GCE for cycles 1–3 (elements D–E), and (C) UA DPV signal with the Pt electrode (element F) is compared to background reactions of PBS at pH 7.4. The A–F annotations refer to the various elements in the SMA. Each curve represents the average of triplicate measurements.
(PNG)

S3 Fig Electrochemical Response of CySH with the SMA Elements. The SMA responses of 60 μM CySH in PBS are shown across the various sensing element. (A) CySH DPV signals at pH 6.5, 7.4 and 8.0 (elements A–C) using a GCE, (B) CySH CV signal at pH 7.4 using GCE for cycles 1–3 (elements D–E), and (C) CySH DPV signal with the Pt electrode (element F) is compared to background reactions of PBS at pH 7.4. The A–F annotations refer to the various elements in the SMA. Each curve represents the average of triplicate measurements.
(PNG)

S4 Fig Electrochemical Signal of CySH/CLZ Simulated Mixture. Differential pulse voltammetry (DPV) of 5.6 μM CLZ, 60 μM CySH, and their mixture in PBS (pH 7.4) at the glassy carbon electrode. This figure demonstrates the additional peaks generated by the cross-reaction of CLZ and CySH. Signal response represents an average of triplicate measurements.
(PNG)

S5 Fig Electrochemical Response of Serum with the SMA Elements. The SMA responses of deproteinized serum are shown across the various sensing element. (A) Serum DPV signals at pH 6.5, 7.4 and 8.0 (elements A–C) using a GCE, (B) serum CV signal at pH 7.4 using GCE for cycles 1–3 (elements D–E), and (C) serum DPV signal with the Pt electrode (element F) is compared to background reactions of PBS at pH 7.4. The A–F annotations refer to the various elements in the SMA, and the proteins were removed from serum samples. Each curve represents the average of duplicate measurements.
(PNG)

S6 Fig Electrochemical Response of CLZ-Spiked Serum with the SMA Elements. The SMA responses of deproteinized serum spiked with 5.6 μM CLZ are shown across the various sensing element. (A) Serum DPV signals at pH 6.5, 7.4 and 8.0 (elements A–C) using a GCE, (B) serum CV signal at pH 7.4 using GCE for cycles 1–3 (elements D–E), and (C) serum DPV signal with the Pt electrode (element F) is compared to background reactions of PBS at pH 7.4. The A–F annotations refer to the various elements in the SMA, and the proteins were removed from serum samples. Each curve represents the average of duplicate measurements.
(PNG)

S7 Fig SMA Response of CLZ-Spiked Serum. Heat map representation of electrochemical responses of the SMA for serum spiked with 5.6 μM CLZ, with signatures highlighted in black outlines. The A-F annotations refer to the various elements in the SMA (Table 1).
(PNG)
