# Supplementary figures and images for: Multidimensional Mapping Method Using an Arrayed Sensing System for Cross-Reactivity Screening
Source: PLoS One. 2015 Mar 19;10(3):e0116310. doi: 10.1371/journal.pone.0116310 (PMC4366158; doi:10.1371/journal.pone.0116310)

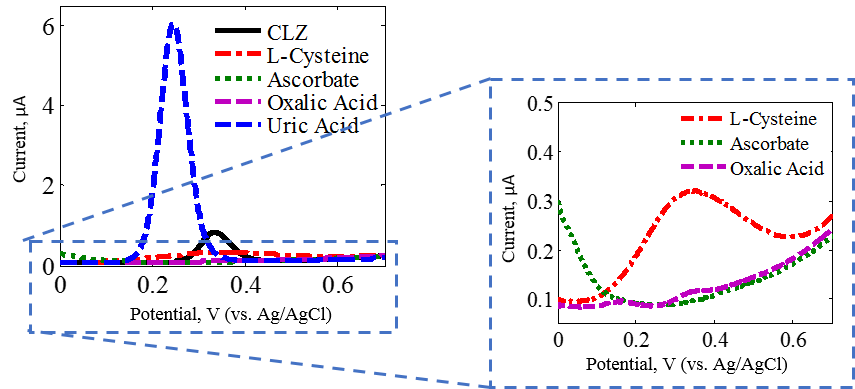

Supplement: S1 Fig — Differential pulse voltammetry (DPV) of 60 μM L-cysteine, 410 μM uric acid, 22 μM oxalic acid, 40 μM ascorbate, and 5.6 μM CLZ (pH 7.4) in PBS at the GCE. Signal response represents an average of triplicate measurements. (TIF) [file pone.0116310.s001.tif]

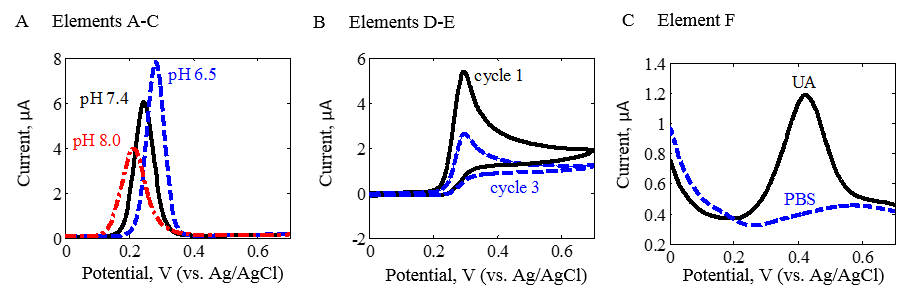

Supplement: S2 Fig — The SMA responses of 410 μM UA in PBS are shown across the various sensing element. (A) UA DPV signals at pH 6.5, 7.4 and 8.0 (elements A–C) using a GCE, (B) UA CV signal at pH 7.4 using GCE for cycles 1–3 (elements D–E), and (C) UA DPV signal with the Pt electrode (element F) is compared to background reactions of PBS at pH 7.4. The A–F annotations refer to the various elements in the SMA. Each curve represents the average of triplicate measurements. (PNG) [file pone.0116310.s002.png]

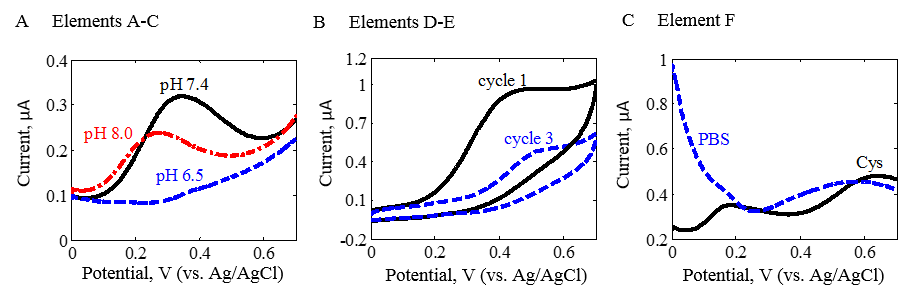

Supplement: S3 Fig — The SMA responses of 60 μM CySH in PBS are shown across the various sensing element. (A) CySH DPV signals at pH 6.5, 7.4 and 8.0 (elements A–C) using a GCE, (B) CySH CV signal at pH 7.4 using GCE for cycles 1–3 (elements D–E), and (C) CySH DPV signal with the Pt electrode (element F) is compared to background reactions of PBS at pH 7.4. The A–F annotations refer to the various elements in the SMA. Each curve represents the average of triplicate measurements. (PNG) [file pone.0116310.s003.png]

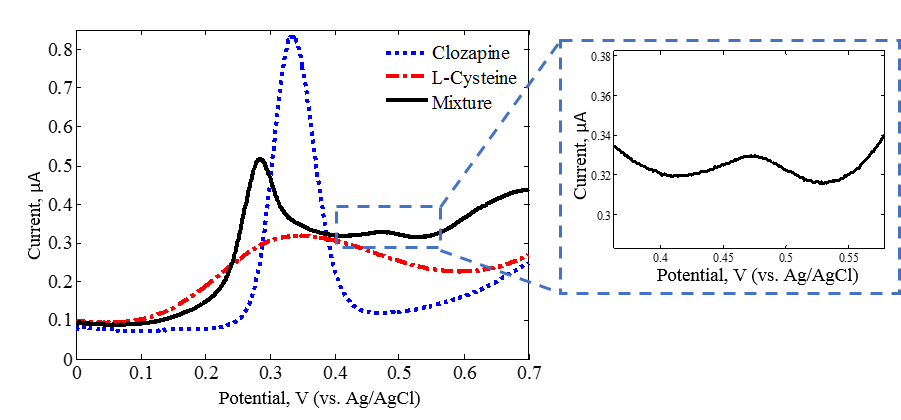

Supplement: S4 Fig — Differential pulse voltammetry (DPV) of 5.6 μM CLZ, 60 μM CySH, and their mixture in PBS (pH 7.4) at the glassy carbon electrode. This figure demonstrates the additional peaks generated by the cross-reaction of CLZ and CySH. Signal response represents an average of triplicate measurements. (PNG) [file pone.0116310.s004.png]

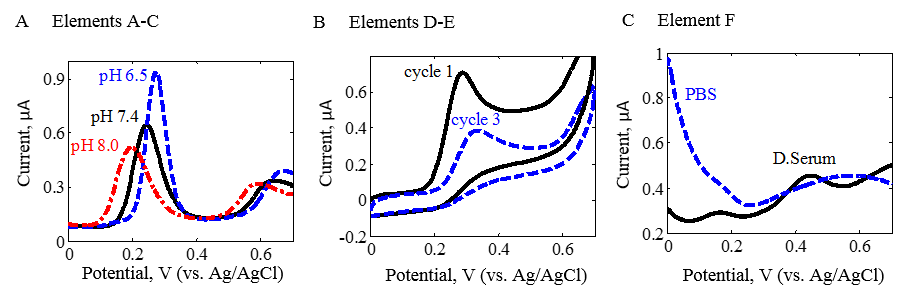

Supplement: S5 Fig — The SMA responses of deproteinized serum are shown across the various sensing element. (A) Serum DPV signals at pH 6.5, 7.4 and 8.0 (elements A–C) using a GCE, (B) serum CV signal at pH 7.4 using GCE for cycles 1–3 (elements D–E), and (C) serum DPV signal with the Pt electrode (element F) is compared to background reactions of PBS at pH 7.4. The A–F annotations refer to the various elements in the SMA, and the proteins were removed from serum samples. Each curve represents the average of duplicate measurements. (PNG) [file pone.0116310.s005.png]

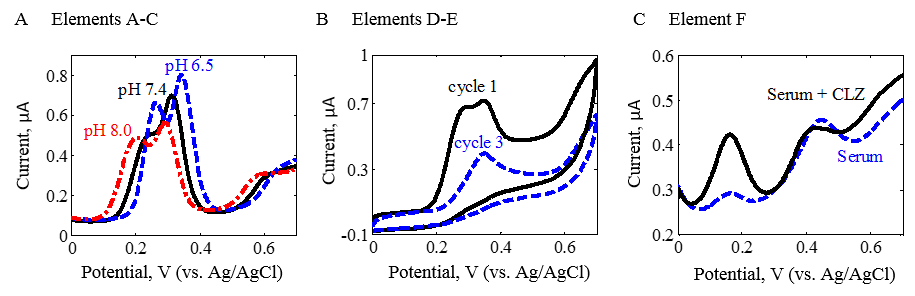

Supplement: S6 Fig — The SMA responses of deproteinized serum spiked with 5.6 μM CLZ are shown across the various sensing element. (A) Serum DPV signals at pH 6.5, 7.4 and 8.0 (elements A–C) using a GCE, (B) serum CV signal at pH 7.4 using GCE for cycles 1–3 (elements D–E), and (C) serum DPV signal with the Pt electrode (element F) is compared to background reactions of PBS at pH 7.4. The A–F annotations refer to the various elements in the SMA, and the proteins were removed from serum samples. Each curve represents the average of duplicate measurements. (PNG) [file pone.0116310.s006.png]

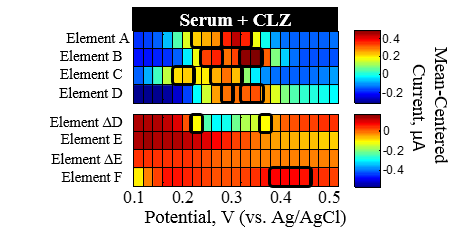

Supplement: S7 Fig — Heat map representation of electrochemical responses of the SMA for serum spiked with 5.6 μM CLZ, with signatures highlighted in black outlines. The A-F annotations refer to the various elements in the SMA (Table 1). (PNG) [file pone.0116310.s007.png]
